# Exploring the role of Luman/CREB3 in regulating decidualization of mice endometrial stromal cells by comparative transcriptomics

**DOI:** 10.1186/s12864-020-6515-2

**Published:** 2020-01-30

**Authors:** Fan Zhao, Huan Liu, Nan Wang, Lijun Yu, Aihua Wang, Yanglei Yi, Yaping Jin

**Affiliations:** 1 College of Veterinary Medicine, Northwest A&F University, Yangling, 712100 Shaanxi China; 2 College of Animal Science and Technology, Northwest A&F University, Yangling, 712100 Shaanxi China; 3 Institute of Biological Resources and Diversity, College of Life Sciences, China Jiliang University, Hangzhou, 310018 Zhejiang China; 4 College of Food Science and Engineering, Northwest A&F University, Yangling, 712100 Shaanxi China

**Keywords:** Luman, RNA-Seq, Transcriptomics, Decidualization, Endometrial stromal cell

## Abstract

**Background:**

Luman is a member of CREB3 (cAMP responsive element-binding) subfamily of the basic leucine-zipper (bZIP) transcription factors. It may play an important regulatory role during the decidualization process since Luman was highly expressed in the decidual cells. However, the exact molecular mechanisms of how Luman regulating decidualization is unknown.

**Results:**

Using an in vitro model, we prove that Luman knockdown significantly affects the decidualization process of mice endometrial stromal cells (ESCs) as the expression of two decidual markers *PRL8a2* and *PRL3c1* were repressed. We employed massively parallel RNA sequencing (RNA-Seq) to understand the changes in the transcriptional landscape associated with knockdown of Luman in ESCs during in vitro decidualization. We found significant dysregulation of genes related to protein processing in the endoplasmic reticulum (ER). Several genes involved in decidualization including bone morphogenetic proteins (e.g. BMP1, BMP4, BMP8A, BMP2, and BMP8B), growth factor-related genes (e.g. VEGFB, FGF10, and FGFR2), and transcription factors (IF4E, IF4A2, WNT4, WNT9A, ETS1, NOTCH1, IRX1, IDB1, IDB2, and IDB3), show altered expression. We also found that the knockdown of Luman is associated with increased expression of cell cycle-related genes including cycA1, cycB1, cycB2, CDK1, CDK2, and PLPK1, which resulted in an increased proportion of ESCs in the G1 phase. Differentially expressed genes (DEGs) were highly enriched on ECM-receptor interaction signaling, endoplasmic reticulum protein processing, focal adhesion, and PI3K-Akt signaling pathways.

**Conclusions:**

Luman knockdown results in widespread gene dysregulation during decidualization of ESCs. Genes involved in protein processing in ER, bone morphogenetic protein, growth factor, and cell cycle progression were identified as particularly important for explaining the decidual deficiency observed in this in vitro model. Therefore, this study provides clues as to the underlying mechanisms that may expand our understanding of gene regulation during decidualization.

## Background

During mammalian reproduction, the uterus undergoes complex processes including implantation, decidualization, and placentation to maintain pregnancy [1]. After the attachment of embryo(s) to the uterine epithelium, the embryo(s) will breach the luminal epithelial barrier and invade into the underlying uterine stroma, which triggers extensive remodeling of the endometrial stromal compartment [2]. This process is known as decidualization, characterized by the differentiation of stromal fibroblasts into specialized decidual cells that coordinate trophoblast invasion and placenta formation [3]. Decidualization plays a critical role in regulating trophoblast invasion, modulating the local immune response at the feto-maternal interface, and supporting the development of the placenta.

Decidualization is driven by the fluctuation of two ovarian steroid hormones, estrogen (E2) and progesterone (P4) [4]. The cellular actions of these hormones are mediated through intracellular estrogen receptor (ER) and progesterone receptor (PR) proteins, which are hormone-inducible transcription factors [5]. Accumulating evidence has demonstrated that endometrial decidualization is associated with a large-scale rewiring of the gene regulatory network, and transcription factors are of great importance in the transcriptional reprogramming during decidualization. The ERα knockout and PR knockout mouse displayed a failure of decidual response to an artificial stimulus, as well as reproductive abnormalities due to defects in multiple reproductive tissues including embryo implantation and uterine decidualization [6, 7]. There are some E2 and P4 regulated transcription factors that control the activation and repression of other gene targets during decidualization. For instance, Hoxa10, a member of the homeobox or Hox multigene family of transcription factors, is required for successful implantation as a partial mediator of P4 signaling [8]. CCAAT/enhancer-binding protein β (C/EBPβ), as a member of the basic leucine zipper (bZIP) family of transcription factors, is able to regulate diverse processes of decidualization. Various studies have shown that C/EBPβ is a key mediator of stromal cell proliferation and decidualization by regulating the expression of multiple cell cycle regulatory proteins [9, 10].

Luman is a member of CREB3 (cAMP-responsive element-binding) subfamily of the basic leucine-zipper (bZIP) transcription factors. Luman was reported to interact with transcriptional coactivator HCF1 [11], hepatitis C virus core protein [12], and dendritic cell-specific transmembrane protein [13]. It involves diverse reproduction-related processes by regulating cell proliferation, differentiation, and apoptosis. In mouse granulosa cells, knockdown of Luman significantly decreases the concentration of E2 and P4 and promote cell proliferation. It was shown that Luman-deficient mice exhibit low corticosterone, low body weight and decreased pup survival [14]. Previous work in our lab revealed that Luman protein was expressed in the luminal, glandular epithelium, and decidual cells during mouse embryo implantation. Moreover, high expression of Luman was detected in the decidualized cells on day 6 to 8 of pregnancy [15], which pinpoint a potential regulatory role of Luman in decidualization.

In this study, we exploited the RNA-seq approach to investigate the role of Luman during mice stromal cell decidualization. Due to the highly dynamic nature of the transcriptome, we monitored the transcriptional changes at 0, 48, and 72 h after in vitro decidualization induction. Additionally, we confirmed our RNA-seq gene expression data by quantitative real-time PCR (qRT-PCR). Finally, the promoter region of the genes belong to a putative Luman regulon was analyzed.

## Results

### Luman expression in ESCs during in vitro decidualization

**Fig. 1:**
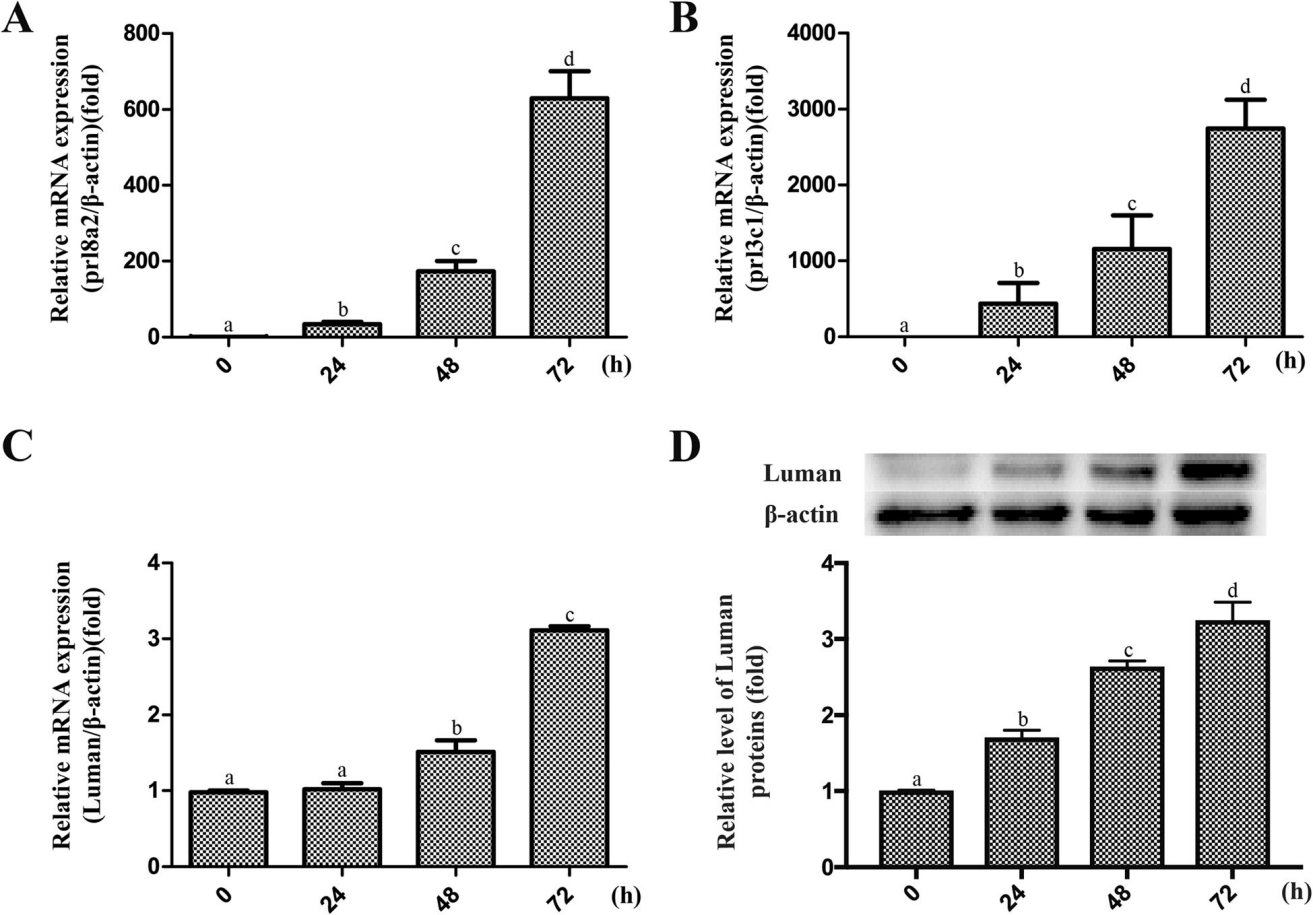
The expression of decidual marker gene and Luman in ESCs during in vitro decidualization. **a** & **b** The mRNA expression of prl8a2 and prl3c1 in ESCs at 0, 24, 48, and 72 h of decidualization. **c** & **d** The mRNA and protein expression of Luman in ESCs at 0, 24, 48, and 72 h of decidualization. *P* < 0.05 was considered significant. All data are represented as the mean ± SEM of repeated experiments (*n* = 3)

Our previous study has shown that Luman protein was expressed in decidual cells of the mouse uterus on Day 6 of pregnancy. The expression level was increased when the second decidual zone was formed, while decreased on Day 8 of pregnancy as apoptosis of the decidualized cells occurred [15]. We further evaluate the Luman expression in ESCs using an in vitro decidualization model. The mRNA expression of two important decidualization markers (*prl8a2* and *prl3c1*) was highly upregulated overtime after the decidual stimulus was added, which implies that the in vitro decidualization model was successfully established (Fig. 1a&b). After 48 and 72 h of decidualization induction, the Luman expression was significantly upregulated at both mRNA and protein level (Fig. 1c&d).

### Luman knockdown decreases the expression of decidualization markers

**Fig. 2:**
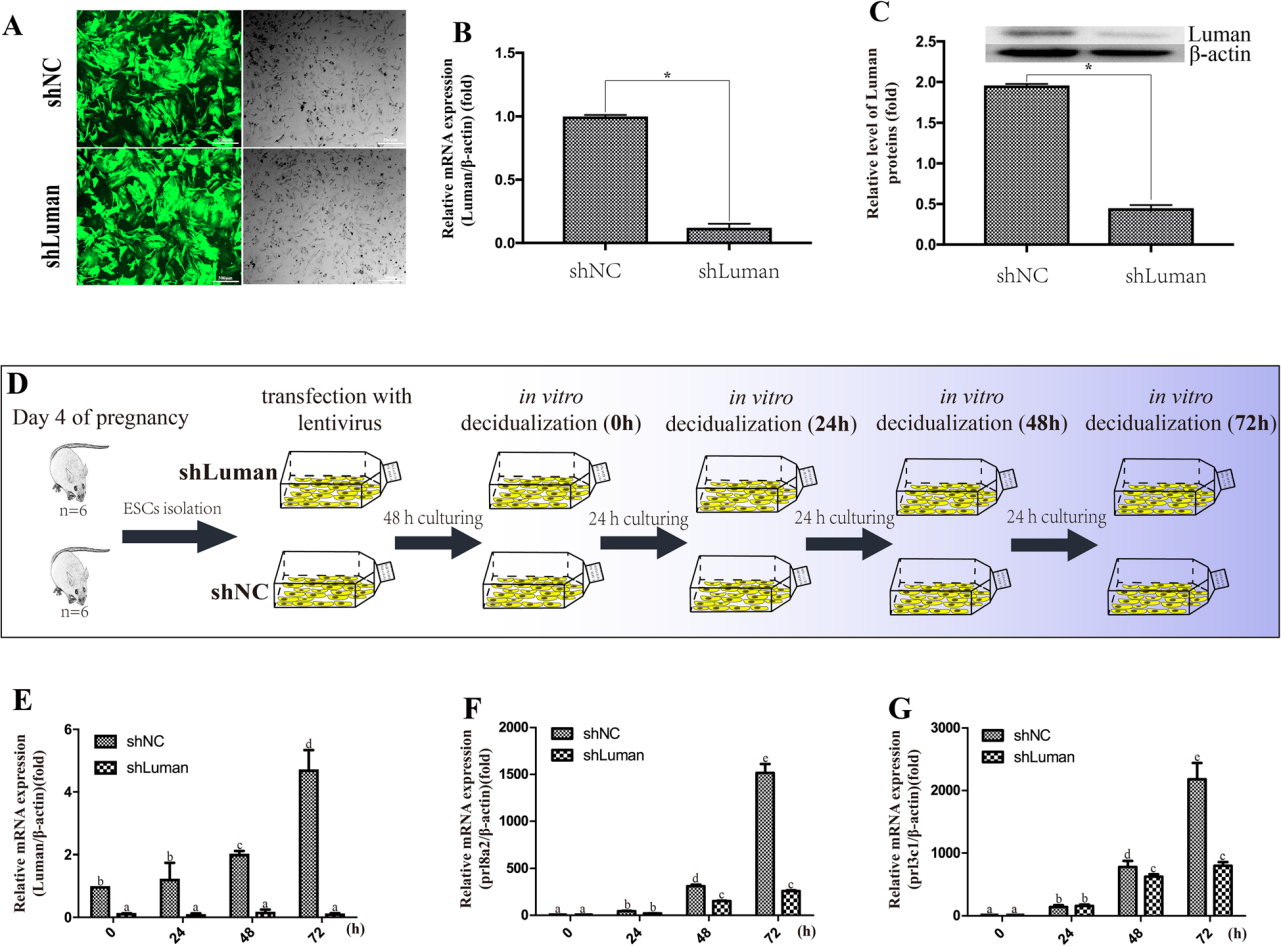
Transfection and knockdown efficiency of shLuman on ESCs and the effects of Luman knockdown on decidualization of ESCs. **a** Transfection efficiency was observed by fluorescence microscopy in the shNC and shLuman transfected ESCs. **b** & **c** The Luman mRNA and protein expression level were detected in the shNC and shLuman groups. **d** The setup with Luman being silenced during different time point after decidualization induction. **e**-**g** mRNA expression of Luman, prl8a2, and prl3c1 in ESCs at 0, 24, 48, and 72 h of decidualization. *P* < 0.05 was considered significant. All data are represented as the mean ± SEM of repeated experiments (*n* = 3)

To determine the role of Luman in ESCs, we transfected the cells with Lentivirus carrying Luman shRNA (shLuman) to block the Luman transcription. The transfected cells showed a strong GFP signal under fluorescent microscopy at 48 h after transfection, indicating a high transfection efficiency of lentivirus (Fig. 2a). After being transfection with shLuman lentivirus, the ESCs showed dramatically decreased mRNA and protein expression of Luman (Fig. 2b & c). We further used this knockdown system to knockdown Luman during in vitro decidualization of ESCs. Cells sampled at 0, 24, 48, and 72 h post decidualization induction were used to analyze the expression of Luman and decidualization markers (Fig. 2d). The results showed that the Luman expression was severely reduced at all sampling point (Fig. 2e). The expression of *prl8a2* and *prl3c1* were significantly downregulated by Luman lentivirus transfection at 48 and 72 h of in vitro decidualization (Fig. 2f & g), indicating an impaired decidualization.

### Luman knockdown leads to distinct Transcriptomic landscapes during in vitro decidualization of ESCs

**Fig. 3:**
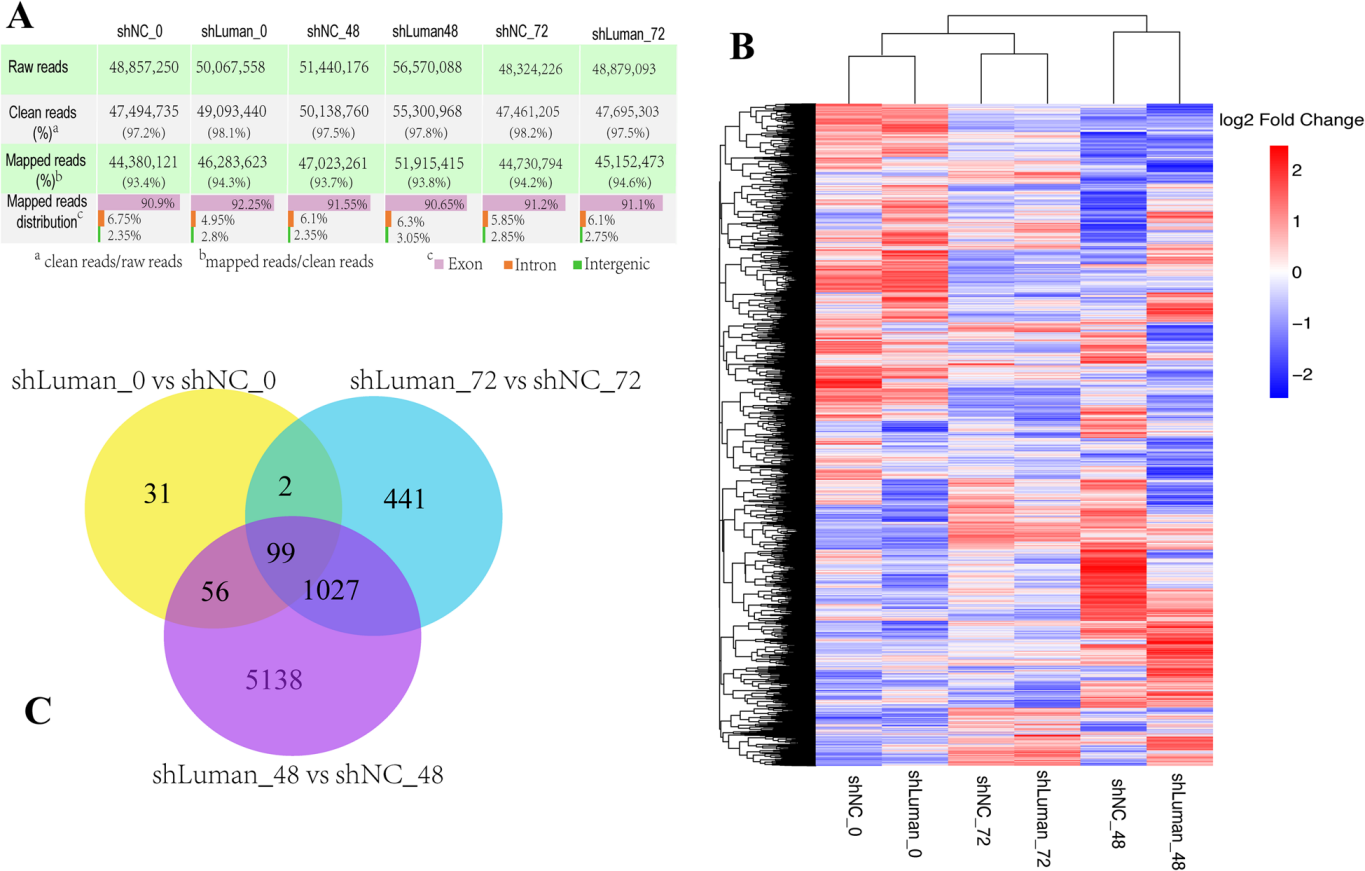
RNA-seq data analysis of in vitro decidualized ESCs in shLuman (Luman knockdown) and shNC (control) group. **a** On average, there are about 50 million reads per library: above 93% of the clean reads aligned to the mouse genome, with about 91% mapped reads located in Exons. **b** Gene expression in different libraries was normalized, centered, and clustered. Blue indicates relatively lower expression while red indicates a higher value. **c** Venn diagram showing the number of genes with altered expression at 0, 48, and 72 h

To study the impact of Luman knockdown on the global transcriptome of ESCs during decidualization, we performed RNA-seq (≈50 million reads per sample) of shLuman- or shNC- lentivirus transfected cells collected at 0, 48, and 72 h after decidualization induction. This is based on our in vitro model that the decidualization was significantly affected by Luman knockdown at 48 and 72 h (Fig. 2f&g). Four technical replicates (individual wells) were pooled into one biological replicate. Two biological replicates were used for each time point. For RNA-seq data analyzing, raw reads were trimmed and aligned to the genome of *Mus musculus*. Reads were then counted and classified. After trimming, more than 95% of the raw reads with good quality (clean reads) were used further analysis. In the mapping step, about 90% clean reads were mapped to exons of the mouse genome, and about 10% clean reads were mapped to introns or intergenic region (Fig. 3a). To compare gene expression, we normalized expression values using DESeq R package (1.18.0) and centered the values. The centered normalized values were visualized as a heat map, which showed obvious clusters of co-expressed genes and clear gene expression differences between Luman knockdown and control group (Fig. 3b). Of the three Luman knockdown groups, ESCs sampled at 0 h of decidualization have least number of differentially expressed genes (188 DEGs), and ESCs sampled at 48 and 72 h of decidualization have 6320 and 1569 DEGs, respectively (Fig. 3c). An additional file shows DEGs in more detail [see Additional file 3].

### Validation of RNA-seq by RT-qPCR

**Fig. 4:**
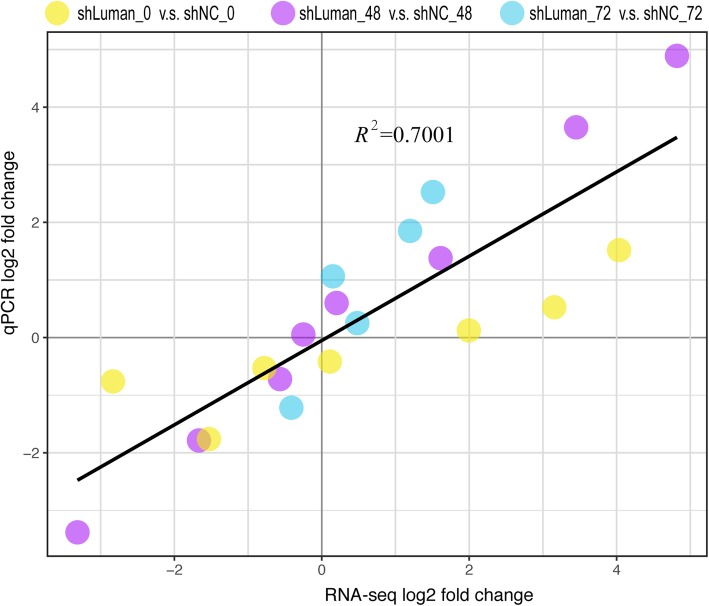
Validation of RNA-seq by RT-qPCR. The study was repeated in duplicates and total RNA was isolated as previously described. Several genes were chosen as validation targets. The fold changes from RT-qPCR against dual RNA-seq foldchanges were plotted with a high degree of correlation

To validate the dual RNA-seq data by RT-qPCR, we chose several genes from three sample time point: seven genes from 0 h (JUND, RS18, PRC2A, AIF1L, CNN2, RN181), eight genes from 48 h (IOD3, ZBT16, LR14B, ATPD, CEBPA, MK67I, LG3BP, BOK), and five genes from 72 h (MMP12, LYZ2, PHYIP, TCTP, PPR3B). The target genes were selected because of their varied expression profiles: increasing, decreasing, or unchanged. The cycle thresholds (Ct) for ESCs transcripts were normalized against *β-actin*. The reference genes were highly expressed and did not show significant changes between any time points during decidualization. The RT-qPCR fold change was calculated by the 2^-ΔΔCt^ method. Fold changes obtained by RT-qPCR and RNA-seq showed a relatively high correlation with R^2^ of 0.7001 (Fig. 4), validating the reliability of the RNA-seq data.

### Luman regulates the ER-associated protein processing during in vitro decidualization of ESCs

**Fig. 5:**
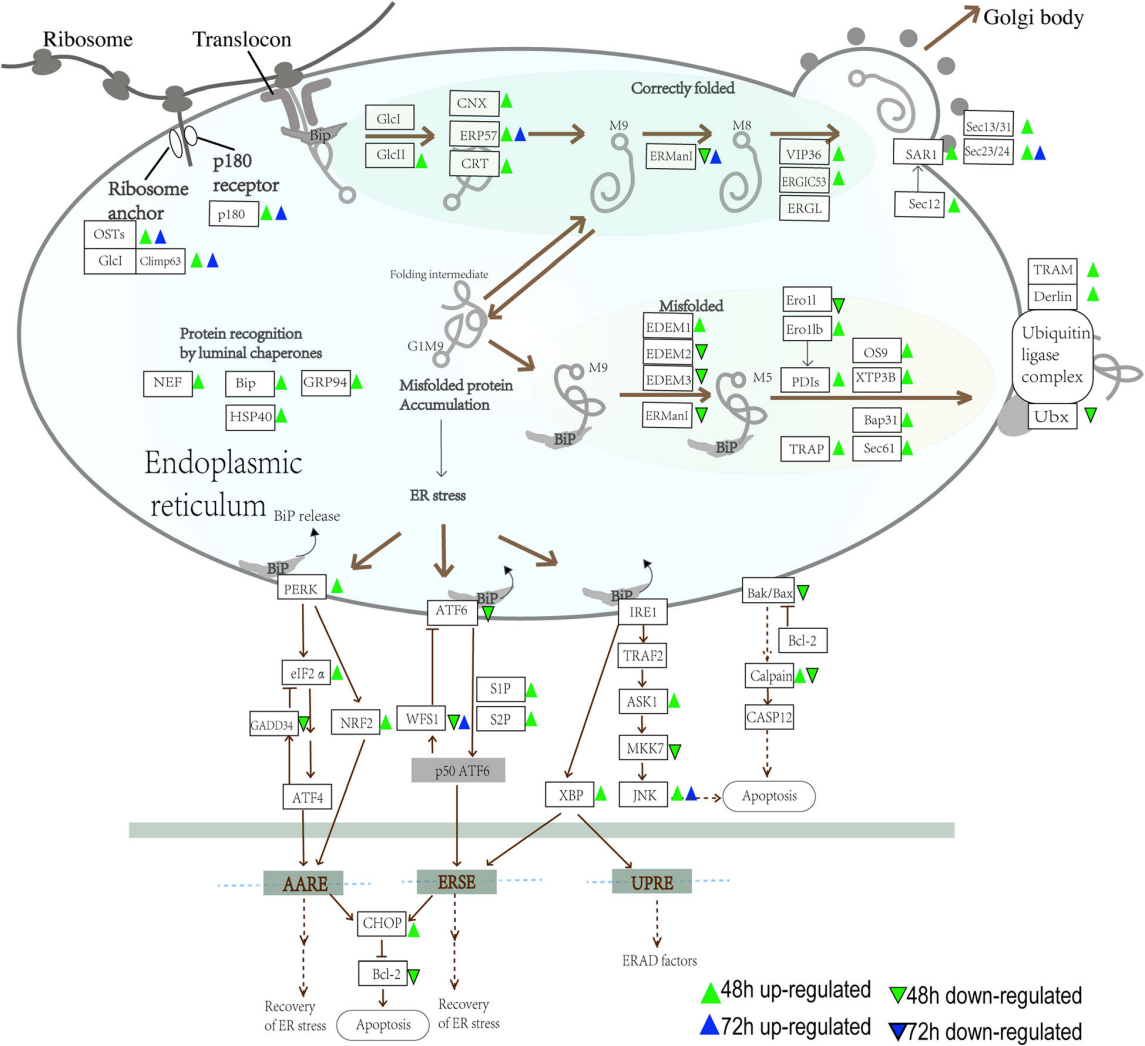
Protein processing pathway in endoplasmic reticulum was affected by Luman knockdown during decidualization. A regular triangle indicates upregulated genes and the inverted triangle indicates downregulated genes

As Luman belongs to the bZIP family whose inactive precursor is localized in the ER membrane, we first examined the genes involved in ER-associated protein processing pathway (Fig. 5). Our results showed that ER-associated genes were prominently affected in Luman-knockdown cells, especially after 48 h of decidualization. The expression of the main ER stress indicator GRP 78 (Bip), as well as its Nucleotide Exchange Factors (NEF, Grp170, and Sil1) [16], are upregulated in of 48 h decidualized ESCs. Another important ER stress chaperone, GRP 94, was also upregulated, indicating an elevated level of unfolded protein in the ER lumen. Endoplasmic reticulum protein folding related genes such as GlcII, CNX, ERP57, and the expression of SAR1 and Sec23/24, which are related to the transport of proteins from the endoplasmic reticulum to the Golgi apparatus, are also upregulated. When the unfolded protein accumulated, part of them enters the ubiquitin-proteasome pathway and eventually being degraded by cytosolic proteasomes. As shown in Fig. 5, the expression of genes involved in the ubiquitin-proteasome pathway including PDIs, TRAP, OS9, and Bap31, etc. were upregulated. In the meantime, the accumulation of misfolded/unfolded proteins within the endoplasmic reticulum (ER) lumen will trigger a signaling pathway named unfolded protein response (UPR) [17]. The UPR signaling activation is also observed in our dataset, as the expression of several genes in the PERK signaling pathway were upregulated.

### Luman regulates the expression of decidualization-related genes

**Fig. 6:**
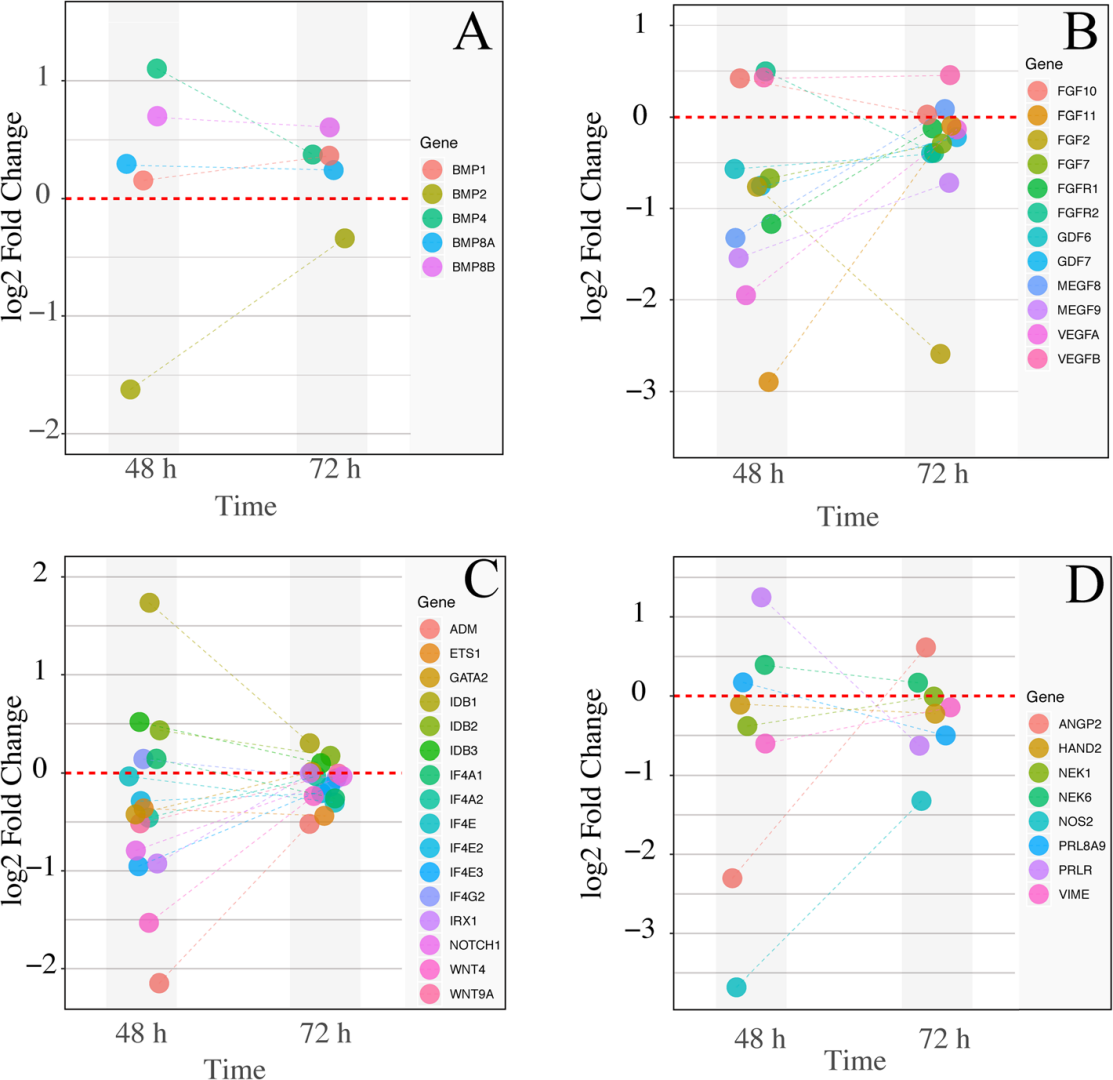
The functionality of decidualization was altered by Luman knockdown. Several genes known to be involved in decidualization were picked and their expression pattern at 48 and 72 h are shown. **a** Genes annotated as bone morphogenetic protein (BMP). **b** Genes annotated as growth factors. **c** Genes annotated as transcription factors. **d** Other genes

To further investigate the effects of Luman knockdown on the decidualization of ESCs, we analyzed several groups of genes that directly related to the decidualization process. In bone morphogenetic proteins (BMP), the expression of BMP1, BMP4, BMP8A, and BMP8B was significantly upregulated at 48 and 72 h of decidualization, while the expression of BMP2 was significantly downregulated at both time points (Fig. 6a). Among the growth factor-related genes, the majority of these genes are down-regulated at 48 and 72 h of decidualization, with VEGFB and FGF10 showed upregulation at both sampling points. FGFR2 was significantly upregulated at 48 h and significantly down-regulated at 72 h of decidualization (Fig. 6b). The expression profile of transcription factors associated with decidualization is relatively complex. Among them, the expression of IF4E, IF4E2, IF4E3, IF4A2, WNT4, WNT9A, ADM, ETS1, NOTCH1, IRX1 gene showed down-regulation at 48 h and 72 h of decidualization, while the expressions of IDB1, IDB2 and IDB3 were up-regulated at both time points (Fig. 6c). In addition, there are some other decidual-related genes showed significantly altered expression after Luman knockdown. For example, the expression of NOS2 is significantly downregulated by Luman at two time points. ANGP2 was significantly down-regulated at 48 h and significantly upregulated at 72 h of decidualization. The trend of PRLR is opposite to that of ANGP2, from a significant upregulation to a significant downregulation (Fig. 6d).

### Effects of Luman knockdown on cell cycle of ESCs during in vitro decidualization

**Fig. 7:**
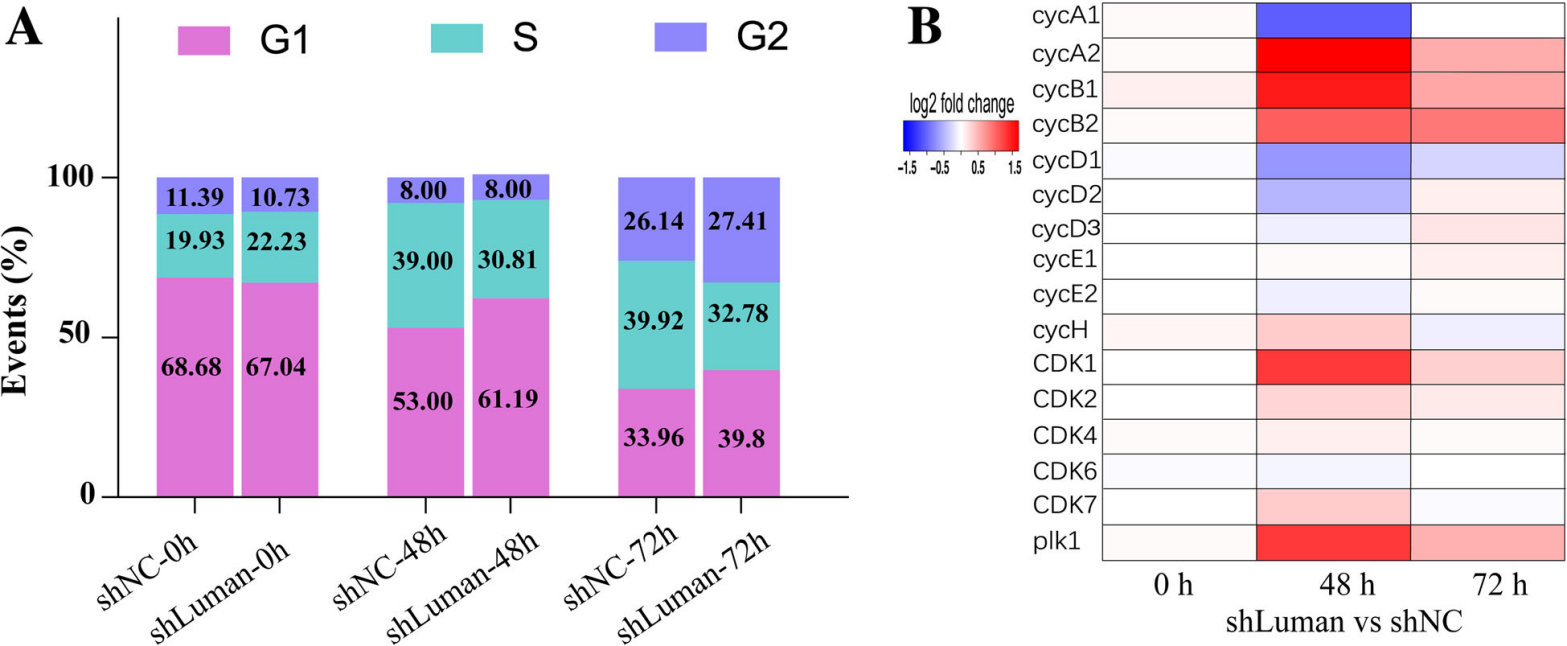
Effects of Luman knockdown on cell cycle of ESCs during decidualization. **a** Analysis of ESCs’ cell cycle by FACS after in vitro decidualization for 48 and 72 h. **b** Expression of cell cycle-associated genes in decidual cells at 48 and 72 h

The cell cycle of decidual ESCs was measured by flow cytometry, and the expression of several important cyclins and their corresponding cyclin-activating enzymes were analyzed. After Luman knockdown, the proportion of ESCs in the G1 phase has increased at both 48 and 72 h of decidualization (Fig. 7a). The RNA-seq data revealed a dramatic expression change of cell cycle-related genes. Cyclin A2 (cycA2), cyclin B1 (cycB1), and cyclin B2 (cycB2) were significantly upregulated at 48 and 72 h of decidualization, while cyclin D1 (cycD1) was significantly downregulated at both time points. The expression of cyclin kinase 1 (CDK1), cyclin kinase 2 (CDK2), and Polo-like kinase 1 (PLK1) was also significantly upregulated at both time points (Fig. 7b).

### Functional validation of representative DEGs

**Fig. 8:**
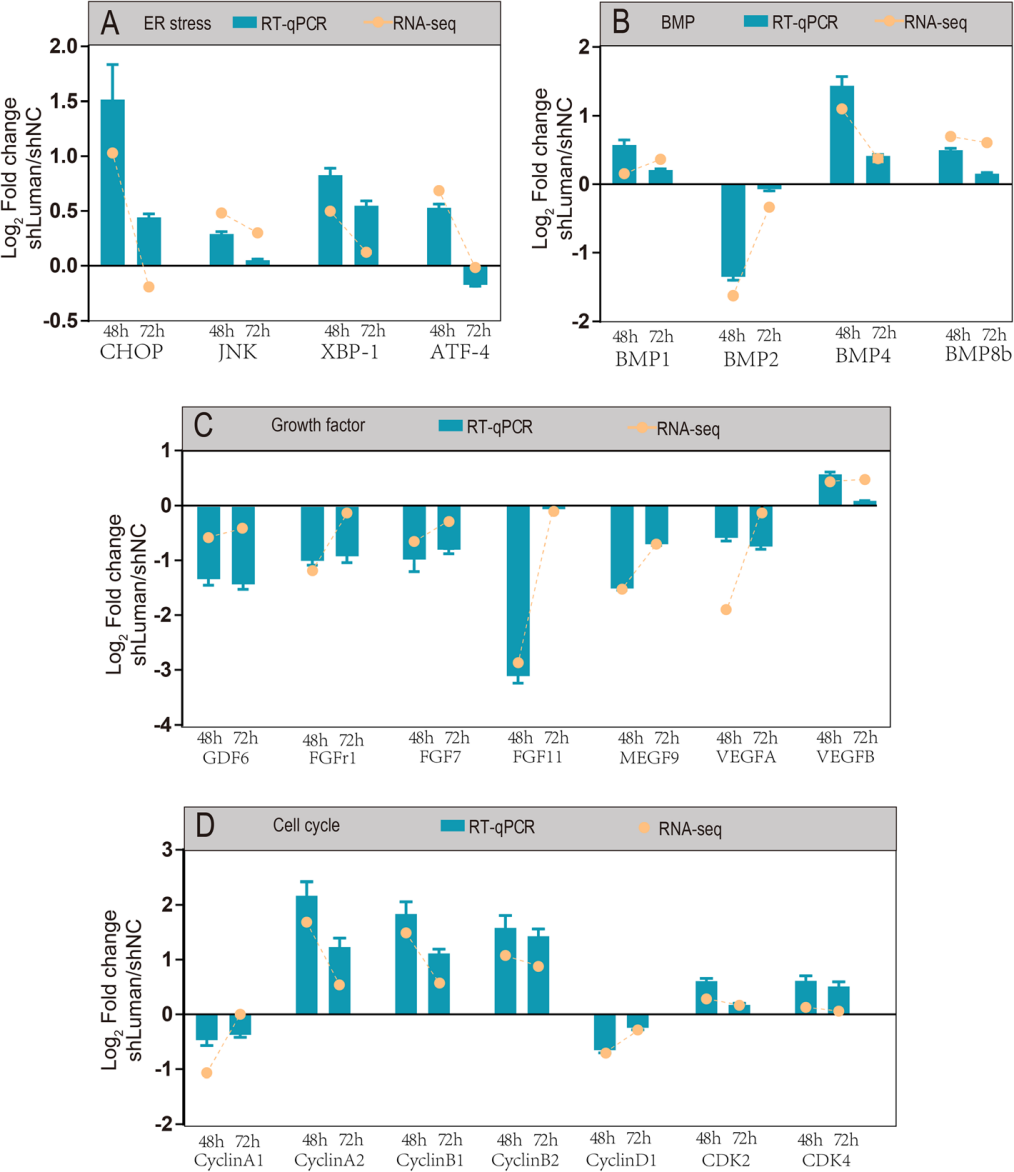
Functional validation of representative DEGs involved in (**a**) ER stress, **b** BMP, **c** Growth factor, and **d** Cell cycle pathways. The histogram shows the RT-qPCR results and the dot indicates the RNA-Seq results

Representative DEGs correlated with ER stress, BMP, growth factor, and cell cycle were selected for functional validation with real-time quantitative PCR assay. Gene expression pattern detected with RT-qPCR was consistent with RNA-seq (Fig. 8). RT-qPCR assay showed that the expression of CHOP was upregulated at 48 and 72 h. The expression level at 72 h was slightly conflicting with the RNA-seq. This can be explained by the fact that most ER stress-related genes were mostly upregualated and CHOP is a well-documented ER stress marker.

### Analysis of the signal pathway of differentially expressed genes (DEGs)

**Fig. 9:**
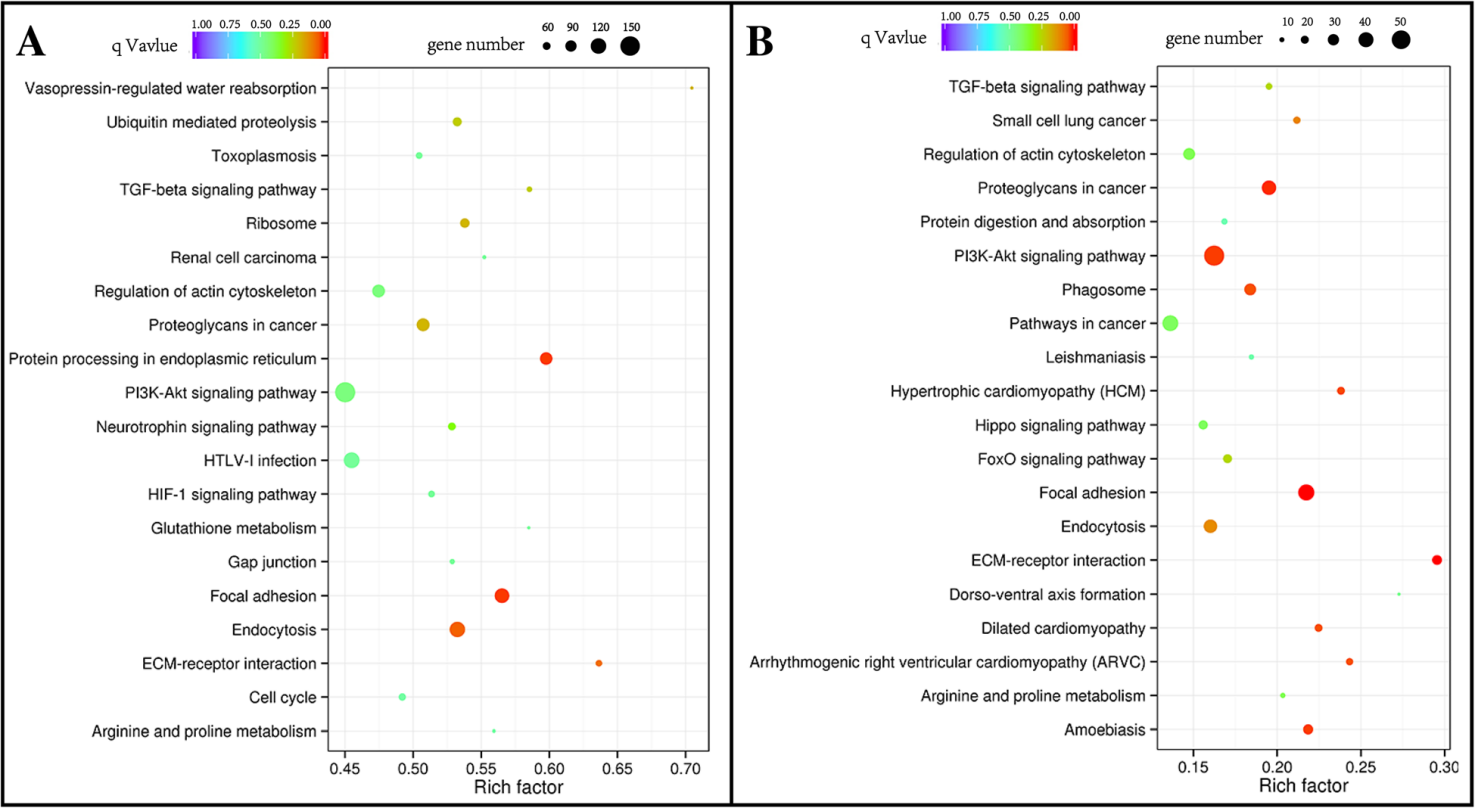
Gene set enrichment analysis and pathway analysis of differentially expressed genes after Luman knockdown in ESCs at (**a**) 48 h and (**b**) 72 h of decidualization

All DEGs were subjected to functional analysis using the KEGG (Kyoto Encyclopedia of Genes and Genomes) database and were categorized according to the pathways involved. An additional file shows this in more detail [see Additional file 4]. The 20 signal pathways with the most enriched genes are shown in Fig. 9. The results showed that the gene enrichment was highest on the ECM-receptor interaction signaling pathway, with 56 and 26 genes (88 genes in the database) being significantly altered at 48 and 72 h of decidualization, respectively. In the 48 h decidual group, the endoplasmic reticulum protein processing was the second most significant pathway. In addition, the focal adhesion and endocytosis pathways also showed significant changes in genes (Fig. 9a). Similar to the 48 h decidual group, the focal adhesion-related genes were also highly enriched in the 72 h decidual group. Moreover, 57 genes were significantly altered in the phosphatidylinositol 3-kinase/Protein Kinase B (PI3K-Akt) signaling pathway, of which 30 genes were up-regulated and 27 genes were down-regulated (Fig. 9b).

### Analysis of Luman regulated genes

**Fig. 10:**
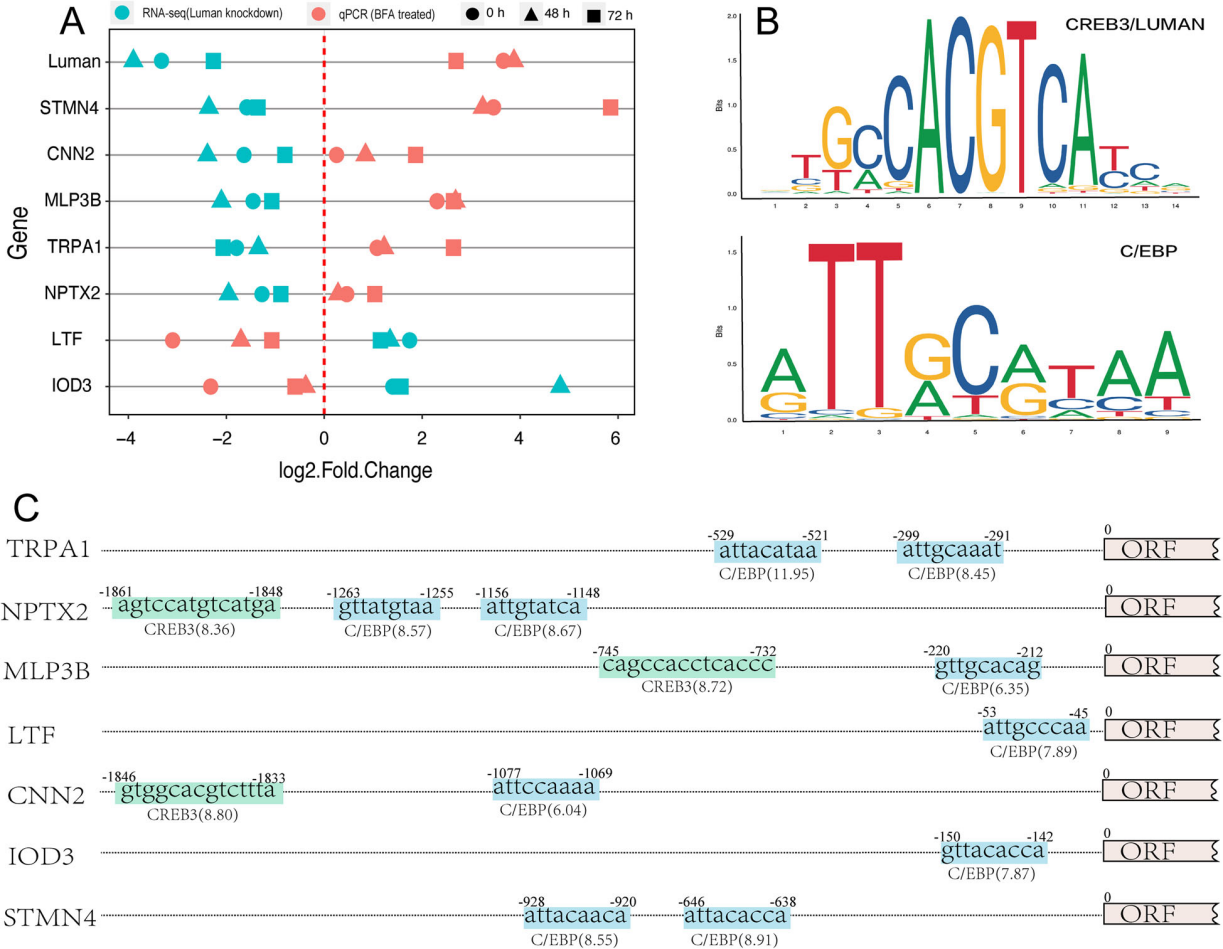
Identification of genes directly regulated by Luman. **a** Gene expression when Luman being downregulated (shLuman) or upregulated (BFA treatment). **b** Weight matrix of two known Luman binding sites. **c** CREB3 and C/EBP conserved binding sites in the promoter region of selected genes

To investigate the genes that directly regulated by Luman, we selected genes that were consistently expressed in RNA-seq data: both up- or down-regulated at three sampling point (0, 48, and 72 h of decidualization). After the addition of the Luman stimulant BFA, the expression of these genes was again determined by RT-qPCR. If the trend at three time points was opposite to the RNA-seq data, the gene is likely to be directly regulated by Luman. As shown in Fig. 10a, the five genes STMN4, CNN2, MLP3B, TRPA1, and NPTX2 were significantly down-regulated at all time points after Luman knockdown, while the two genes TRFL and IOD3 were significantly upregulated at three time points. After treatment of ESCs with BFA, Luman expression was significantly upregulated. At the same time, the expression of STMN4, CNN2, MLP3B, TRPA1, NPTX2 was upregulated, while the expression of TRFL and IOD3 was significantly downregulated. Previous studies have demonstrated that Luman regulates the expression of downstream genes by binding to specific sites in the promoter region. Known Luman regulatory sites include CREB3 and C/EBP conserved sequences [18], the sequences of which are shown in Fig. 10b and the letter size indicates the probability of occurrence of a particular base at that site. By analyzing the promoter sequences of the above seven genes, it was found that the C/EBP binding sequence was detected in the promoter regions of all genes, and the direct binding sequence of Luman was detected in the promoter regions of NPTX2, MLP3B, and CNN2 genes. These genes may be directly regulated by Luman and play an important role in the decidualization process (Fig. 10c).

## Discussion

This study applied RNA-seq to investigate the effects of Luman knockdown on the gene expression of decidualized ESCs based on an in vitro decidualization model. In this model, the effect of Luman knockdown on two decidual markers prl8a2 and prl3c1 began to appear after 48 h of decidualization induction, and the effect was more significantly inhibited after 72 h of induction. Therefore, cells at these two points were collected for RNA-seq analysis. The results show that the gene expression of the decidualized 0-h cells was not significantly affected by Luman, but was significantly affected by Luman at 48 and 72 h of decidualization.

As a transcription factor localized in the endoplasmic reticulum membrane and a potential ER stress sensor, Luman knockdown significantly increases the level of unfolded protein and therefore affects the expression of ER protein processing-related genes. Sequentially, the expression of key genes of ER-associated protein degradation (ERAD) pathway was significantly upregulated. Termine et al. reported that the transcriptional elevation of EDEM1 boosts the efficiency of ERAD through the formation of a complex that suppresses the proteolytic downregulation of ER mannosidase I (ERManI) [19], which was also verified in this study. OS-9 and XTP3B are capable of binding to misfolded proteins and link ERAD substrates to the membrane-associated ubiquitination machinery [20]. In this study, the expression levels of OS-9 and XTP3B were significantly up-regulated, indicating that Luman knockdown increases the activity of ERAD and related ubiquitination degradation pathway. Moreover, the ER stress receptor IRE1 and its downstream genes were up-regulated, suggesting that Luman knockdown causes unfolded protein responses reaction in cells.

Luman knockdown affects the expression of a wide range of genes related to decidualization. The expression of several bone morphogenetic proteins (BMP) was altered. Among them, BMP2 was significantly downregulated, while BMP1, BMP4, and BMP8 were significantly upregulated at 48 h and 72 h of decidualization. BMP2 has been reported as a key regulator of gene expression and function in mouse uterine decidualization [21]. The role of BMP1, BMP4, and BMP8 in decidualization has not been reported, and the specific mechanism needs further study. The expression of many growth factor-related genes was down-regulated in both 48 and 72 h decidualized ESCs. These genes play important roles in decidualization or cell growth and differentiation. For instance, FGF7 and FGFR2 can stimulate the proliferation of human ESCs and the expression of insulin-like growth factor-binding protein 1 and prolactin in an autocrine manner through the PERK and JNK signaling pathways [22]. FGF-10 and FGFR-1 have key roles in decidual-trophoblast interaction [23]. GDF7 is important for interneurons and sensory neurons, as well as for the development of seminal vesicles [24]. Luman’s knockdown led to a significant down-regulation of WNT4, which is a critical regulator not only of proper postnatal uterine development but also embryo implantation and decidualization [25]. Moreover, down-regulation of Nothc1 and NOS2 may have a direct effect on decidualization. The Notch1 signaling is involved in inhibiting apoptosis of stromal fibroblasts and regulate cell cycle progression prior to embryo implantation, thereby ensuring the success of decidualization [26]. NOS_2_ plays an important role in maintaining the integrity of the decidual cells and the normal development of the uterine vascular system and may have the effect of promoting the survival of the embryo [27].

The signal pathway enrichment analysis showed that the extracellular matrix-receptor interaction (ECM-receptor interaction) signaling pathway enriched most DEGs after Luman knockdown. The decidual stromal matrix undergoes extensive reorganization beginning at the peri-implantation period since the extracellular matrix (ECM) provides mechanical support for the epithelium, stroma, and vessels during decidualization [28]. The transcriptomic study on decidualization of rat stromal cells revealed that the ECM-related genes were significantly up-regulated [29]. During the decidualization process, the structure and molecular composition of the endometrial extracellular matrix (ECM) undergone significant changes [30]. The focal adhesion signaling pathway is also significantly enriched. It is reported that the focal adhesion kinase on decidual cells is important in development and differentiation following attachment [31]. In rats, focal adhesion-associated proteins are involved in the invasion of the blastocyst into the endometrial decidual cells [32]. In the 72 h decidual group, DEGs were significantly enriched in the PI3K/Akt signaling pathway. Fabi and coworkers found that Akt expression is down-regulated in human ESCs, and the inhibition of the PI3K pathway may be involved in decreased cell motility during decidualization [33]. Similarly, knockdown of non-metastasis gene 23 (nm23) in ESCs of mouse and human decreased cell proliferation and decidualization through the PI3K/Akt signaling pathway [34].

A group of genes shows consistent expression patterns in the 0, 48, and 72 h decidual groups after Luman knockdown. We speculate that those genes are directly regulated by Luman. Furthermore, when ESCs were treated by BFA (to elevate Luman expression), high consistency in mRNA expression of STMN4, CNN2, MLP3B, TRPA1, NPTX2, TRFL and IOD3 was observed again. Lu et al. reported that Luman control gene transcription by binding to certain conserved sites in the promoter region of the downstream genes. These sites include CREB (Luman) or C/EBP conserved sequence [18]. Of the genes with similar expression pattern, we found at least one Luman-binding site in their promoter region, which further prove those genes are potentially belong to Luman regulon.

## Conclusions

In summary, Luman plays an important role in the decidualization of ESCs. Knockdown of Luman causes an increase in unfolded proteins in the endoplasmic reticulum, affecting stromal cell cycle and the expression of decidual-related genes. These regulatory effects are associated with the endoplasmic reticulum signaling pathway, the extracellular matrix, and the PI3K/Akt signaling pathway.

## Methods

### Animal

Female adult Kunming White mice (SPF grade, 7–9 weeks old) were purchased from Experimental Animal Center of The Fourth Military Medical University. All the mice were caged in a controlled environment with a cycle of 12 L:12D at 22 °C. All procedures were approved by the Committee for the Ethics on Animal Care and Experiments in Northwest A&F University.

### ESCs isolation and in vitro decidualization induction

To set up the matings, female mice in estrus are placed into the cages with males (two females in each cage with one male). In the next morning, females were checked for vaginal plugs for successful mating. A total of twelve female mice were used for the study (shLuman, *n* = 6; shNC, *n* = 6). To minimize suffering during sacrifice, mice on day 4 of pregnancy were euthanized by cervical dislocation. ESCs were isolated from fresh endometrial tissue and were cultured at 37 °C with 5% CO_2_. The epithelial and stromal cells were separated by the adhesion purification method (see Additional file 1). In vitro decidualization induction was performed as previously described [35]. Briefly, the confluence ESCs from the third passage after thaw were treated with 10 nM of estradiol-17b (E2; Sigma-Aldrich, Germany) and 1 μM of progesterone (P4; Sigma-Aldrich, Germany) to induce decidualization. Culture medium was changed every 2 days. Cells were observed and photographed with an inverted microscope during the treatment period (see Additional file 2).

### Transfection of ESCs with shLuman lentivirus

Lentivirus vectors bearing the Luman shRNA sequence (shLuman) and the non-silencing sequence (shNC) were constructed previously [36]. shLuman lentivirus was packaged as described by *Chen* et al [37]. The supernatant of cell culture containing shLuman or shNC lentivirus was stored at − 80 °C. One day prior transfection, about 2 × 10^5^ ESCs were seeded into 6-well plate with 50–60% confluence. The complete culture solution was replaced by 2 ml lentiviral particles with 2 μl polybrene (8 mg/ml; GeneChem Co., Ltd., China). After 12 h, the lentivirus solution was replaced by complete culture medium and cultured for 48 h. Cells were collected for downstream experiments.

### RNA isolation, sequencing, and data analysis

ESCs were transfected by Lentivirus carrying either Luman shRNA sequence (shLuman) or the non-silencing sequence (shNC). Cells were cultured for 48 h to allow Luman knockdown, decidual stimulus was then added and was designated as 0 h post decidualization. Samples were taken at 0, 48, and 72 h post decidualization from shLuman or shNC group. Six technical replicates (individual wells) were pooled into one biological replicate. Two biological replicates were used for each time point. Total RNA was isolated from ESCs with the TRIzol reagent (TaKaRa Bio Inc., Japan), RNA quantity and purity were determined by a NanoDrop spectrophotometer and 1% agarose gel. The RNA integrity was assessed using the RNA Nano 6000 Assay Kit of the Bioanalyzer 2100 system (Agilent Technologies, CA, USA).

A total amount of 3 μg RNA per sample was used as input material for the RNA sample preparations. NEBNext® Ultra™ RNA Library Prep Kit for Illumina® (NEB, USA) was used to prepare sequencing libraries following the manufacturer’s recommendations. Index codes were added to attribute sequences to each sample. cDNA fragments of preferentially 250~300 bp in length were obtained by purifying the library fragments with AMPure XP system (Beckman Coulter, Beverly, USA). After PCR amplification, the products were purified (AMPure XP system) and library quality was assessed on the Agilent Bioanalyzer 2100 system. The library preparations were sequenced on an Illumina Hiseq platform and 125 bp/150 bp paired-end reads were generated.

Raw reads were processed through in-house Perl scripts to remove reads containing adapter, reads containing poly-N, and reads of low quality. At the same time, Q20, Q30 and GC content of the clean data were calculated. All the downstream analyses were based on clean data with high quality. Reference genome and gene model annotation files were downloaded from the NCBI website directly. Index of the reference genome was built using Bowtie v2.2.3 and paired-end clean reads were aligned to the reference genome using TopHat v2.0.12. HTSeq v0.6.1 was used to count the reads numbers mapped to each gene. And then FPKM of each gene was calculated based on the length of the gene and reads count mapped to this gene. Differential expression analysis of two conditions/groups (two biological replicates per condition) was performed using the DESeq R package (1.18.0). Genes with an adjusted *P*-value < 0.05 found by DESeq were assigned as differentially expressed. The sequence reads of this study have been submitted to the NCBI Sequence Read Archive (SRA) under accession number SRP216935.

### RT-qPCR

One microgram of total RNA was treated with DNase-I (TaKaRa Bio Inc., Japan) and subjected for cDNA synthesizing using the PrimeScript™ RT reagent Kit (TaKaRa Bio Inc., Japan). RT-qPCR was performed with a LightCycler system (iQ5, Bio-Rad, USA). Each reaction was performed in a total 20 μl reaction system containing 10 μl 2 × SYBR® Premix Ex Taq™II(Tli RNaseH Plus, TaKaRa Bio Inc., Japan), 2 μl cDNA, 0.8 μl of each primer (10 μM) and 6.4 μl of nuclease-free water. RT-qPCR primers were designed using PerlPrimer (http://perlprimer.sourceforge.net/) software across intron/exon boundaries. Primer sequences are listed in Additional file 5 and *β-actin* gene was included as a housekeeping gene. Moreover, melting curve analysis was performed after real-time PCR reactions to monitor PCR product specificity. At least three biological replicates were performed for each sample. Relative fold change was analyzed according to the 2^-△△Ct^ method against housekeeping gene (*β-actin*).

### Western blot analysis

The total protein isolation and protein quantification were performed as previously described [36]. The protein samples were stored at − 80 °C for subsequent use. For SDS-PAGE, the samples were separated on 12% polyacrylamide gel and transferred to PVDF membranes (Millipore, Bedford, MA). The membranes were blocked for 1 h in 10% skimmed milk diluted in Tris-buffered saline (TBS) and incubation for 1 h at Room temperature with primary antibodies: anti-Luman (1:400, made by our laboratory, see reference for details [15]) or anti-β-actin (1:1000, Tianjin Sanjian Biotech Co., Ltd., Tianjin, China). After being washed three times with TBS containing 0.1% Tween-20, membranes were incubated with corresponding secondary antibody conjugated to HRP (1:2000, Zhongshan Golden Bridge Biotechnology, China) for 1 h at room temperature. Finally, the bands were visualized utilizing Gel Imaging System (Tannon Science & Technology Co. Ltd., China) and then analyzed with the Quantity One software (Bio-rad, USA).

### Cell cycle analysis

Cell cycle analysis was performed as previously described [36]. Briefly, transfected ESCs (2 × 10^5^ cells/well in 6-well plate) were washed with PBS and fixed in ice-cold 70% ethanol overnight at 4 °C. Then, cells were stained with propidium iodide/RNase A solution at 37 °C for 30 min in a dark chamber. Flow cytometric analyses were conducted using a BD FACS Calibur system. For each determination, a minimum of 20,000 cells was analyzed. All experiments were repeated three times.

### BFA treatment and promoter analysis

We hypothesized that genes with consistent expression changes at 0, 48, and 72 h of decidualization possibly have conserved Luman-binding sites in their promoter region. To prove this hypothesis, we treated the cells with brefeldin A (BFA, 1 μg/mL) for 18 h to up-regulate the expression of Luman. Cells were decidualized for 0, 48, 72 h, and then RNA was extracted and subjected to RT-qPCR analysis. Genes with same expression pattern in Luman up-regulated (BFA treatment) or down-regulated (Luman knockdown) group were identified as potential Luman-regulated genes, whose promoter sequence were analyzed for Luman-binding sites by using the JASPAR [38] database.

## Supplementary information

**Additional file 1.** Morphology of mouse primary endometrial stromal cells (arrows indicate mouse endometrial stromal cells). (A) Non-adherent endometrial stromal cells and other impurity cells that have just been isolated; (B) Endometrial stromal cells and impurities after 2 h of culture; (C) After 24 h of culture, mostly mouse endometrial stromal cells have irregular prismatic or triangle shape.

**Additional file 2.** In vitro decidualization induction of endometrial stromal cells. (A) Endometrial stromal cells were adhered for 24 h after isolation and were recorded as in vitro decidualization induction for 0 h. The cell was small with good translucency, and the shape showed irregular rhomboid or triangle. (B) In vitro decidualization induction for 24 h. The cell size began to increase, and the morphology becomes irregular. (C) In vitro decidualization induction for 48 h. The cell size became bigger, the cell showed a long fusiform shape with reduced translucency. (D) In vitro decidualization induction for 72 h, the cell volume continued to increase and the cell boundaries were blurred.

**Additional file 3.** Illumina RNA-Seq data of all significantly differentially expressed genes in different categories (Sheet 1, shLuman vs shNC at 0 h decidualization; Sheet 2, shLuman vs shNC at 48 h decidualization; Sheet 3, shLuman vs shNC at 72 h decidualization).

**Additional file 4.** List of differentially expressed genes enriched in different signal pathways. (Sheet 1, shLuman vs shNC at 48 h decidualization; Sheet 2, shLuman vs shNC at 72 h decidualization).

**Additional file 5.** Sequences of primer pairs for RT- qPCR.

## Data Availability

The datasets supporting the conclusions of this article are included within the article and Additional files 1, 2, 3, 4, 5. Complete RNA-seq datasets are available from NCBI (accession NO. SRP216935).

## Abbreviations

BFA: Brefeldin A
CREB: cAMP responsive element-binding
DEGs: Differentially expressed genes
E2: estrogen
ERAD: ER-associated protein degradation
ESC: Endometrial stromal cell
FPKM: Fragments Per Kilobase of transcript sequence per Millions base pairs sequenced
KEGG: Kyoto Encyclopedia of Genes and Genomes
P4: progesterone
UPR: Unfolded protein response
