# Supplementary figures and images for: Exploring the role of Luman/CREB3 in regulating decidualization of mice endometrial stromal cells by comparative transcriptomics
Source: BMC Genomics. 2020 Jan 30;21:103. doi: 10.1186/s12864-020-6515-2 (PMC6993373; doi:10.1186/s12864-020-6515-2)

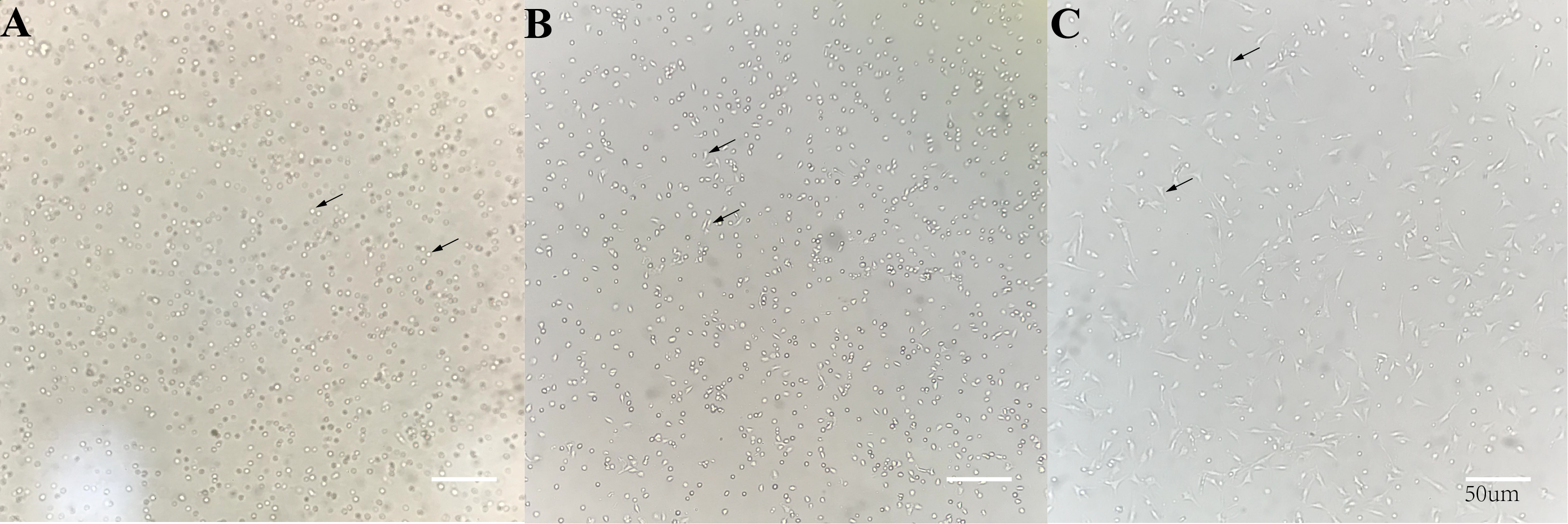

Supplement: Supplementary file 1 — Additional file 1. Morphology of mouse primary endometrial stromal cells (arrows indicate mouse endometrial stromal cells). (A) Non-adherent endometrial stromal cells and other impurity cells that have just been isolated; (B) Endometrial stromal cells and impurities after 2 h of culture; (C) After 24 h of culture, mostly mouse endometrial stromal cells have irregular prismatic or triangle shape. [file 12864_2020_6515_MOESM1_ESM.tif]

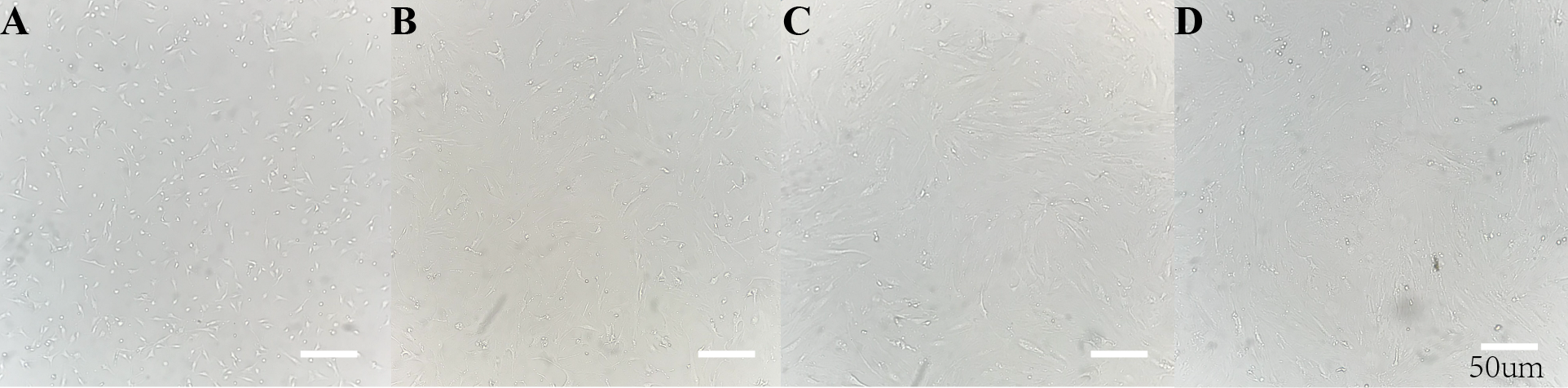

Supplement: Supplementary file 2 — Additional file 2. In vitro decidualization induction of endometrial stromal cells. (A) Endometrial stromal cells were adhered for 24 h after isolation and were recorded as in vitro decidualization induction for 0 h. The cell was small with good translucency, and the shape showed irregular rhomboid or triangle. (B) In vitro decidualization induction for 24 h. The cell size began to increase, and the morphology becomes irregular. (C) In vitro decidualization induction for 48 h. The cell size became bigger, the cell showed a long fusiform shape with reduced translucency. (D) In vitro decidualization induction for 72 h, the cell volume continued to increase and the cell boundaries were blurred. [file 12864_2020_6515_MOESM2_ESM.tif]
