# Supplementary material for: Exploring the role of Luman/CREB3 in regulating decidualization of mice endometrial stromal cells by comparative transcriptomics
Source: BMC Genomics. 2020 Jan 30;21:103. doi: 10.1186/s12864-020-6515-2 (PMC6993373; doi:10.1186/s12864-020-6515-2)
Supplement: Supplementary file 5 — Additional file 5. Sequences of primer pairs for RT- qPCR. [file 12864_2020_6515_MOESM5_ESM.docx]

**Table S1** Sequences of primer pairs for qRT-PCR.

| Target  gene | GenBank accession No. | Forward (5′-3′) | Reverse (5′-3′) |
| --- | --- | --- | --- |
| β-actin | NM_007393 | GCAAGCAGGAGTACGATGAG | CCATGCCAATGTT GTCTCTT |
| Prl8a2 | NM_010088 | AGCCAGAAATCACTGCCACT | TGATCCATGCACCCATAAAA |
| Prl3c1 | NM_001163218 | GCCACACGATATGACCGGAA | GGTTTGGCACATCTTGGTGTT |
| MMP12 | NM_001320076.1 | ACTACTGGAGGTATGATGTGAG | TAGTTACACCCTGAGCATAGAG |
| LYZ2 | NM_017372.3 | TCAATTGCAGTGCTCTGCTG | GCCCTGTTTCTGCTGAAGTC |
| PHYIP | NM_145981.3 | GTGACTCTTTCCGTATCTCCT | AACTCCACAGTCTCACTCCA |
| TCTP | NM_145981.3 | ATCTACCGGGACCTCATCAG | GTTCTACTGACCATCTTGCCC |
| CNN2 | NM_007725.2 | GGCTCCTGTCCAAATATGACC | CTTGTTCATGAGTGTGCATAGG |
| RS18 | NM_011296.2 | AATAGCCTTCGCCATCACTG | GGATCTTGTACTGTCGTGGG |
| CCDC127 | NM_001168658.1 | CAGCATTTCGTTGGATTTGG | CTTTATCTGAGCCCGTTCCT |
| LG3BP | NM_011150.2 | GGACTCAAGGTACAGAAGATGGA | ACATTCCACCTCATCCAGCA |
| AIF1L | NM_145144.1 | TTTCCTCCCTGTTGTCCCTG | AAAGACATCAGATCAATCTCGCC |
| CEBPA | NM_001287523.1 | CTCTGATTCTTGCCAAACTGAG | GACCCACTACTACATACACCC |
| ATPD | XM_017314052.1 | TCACTAGGGTTCCTTTATGGGTC | TTGGGTTCGGAAGTCTATCCTG |
| IOD3 | NM_172119.2 | CACCATCATGTACCAGGGAG | CCACCAATTCAGTCACTTGTC |
| JUND | NM_001286944.1 | ACACTTGGGAATATGAAACAGG | GAAACTGAGGATGGGATGTG |
| BOK | NM_016778.3 | CTTCTCAGCAGGTATCACATGG | GGTCTTGCGTACAAACTCCC |
| MK67I | NM_026472.4 | TTCCCTCACTGGTCCTACCT | GTCCACAGACGACTTCTTCCT |
| ZBT16 | NM_001364543.1 | CACATACAGGTGACCACCCA | ATGGTACACTGGTATGGCGA |
| PRC2A | NM_020027.3 | GTTTACATACTGCCACAAGCC | GACTGACTCATCTCTGTGCC |
| RN181 | XM_006506478.2 | CTGACTTATCCAACCAACGAG | CAGGAATTTGTCTTACTTAGCCAG |
| PPR3B | XM_017312797.1 | CAATCAACCCTGACACTTCTC | GCTTCTTCACCTTCTTCTCC |
| TRPA1 | XM_006495526.2 | ATGAACTTACTGATTGGCTTGG | GGTCTATTTGGATACACGATGG |
| NPTX2 | NM_016789.3 | CAAATGTGTCTAACGCTGGG | TAGTTTGTACGGAGAGGAAGG |
| MLP3B | NM_026160.4 | AGATCCCAGTGATTATAGAGCGA | ATTGCTGTCCCGAATGTCTC |
| STMN4 | XM_006519316.2 | GTGCCTGAGTTTAATGCCTCC | TTCCTGGTACTTCCTTCGCT |
| LTF | NM_008522.3 | AGAATACTGACGGGAAGAACAC | AACTCTCCTGGACACCTCTG |
| CHOP | NM_007837.3 | AGCTGGAAGCCTGGTATGAGGA | AGCTAGGGACGCAGGGTCAA |
| JNK | XM_006519032.4 | CCGGACAAGCAGTTAGATG | CACCTGTGCTAAAGGAGAC |
| XBP-1 | NM_001271730 | GAGCAGCAAGTGGTGGATTT | AAAGGGAGGCTGGTAAGGAA |
| ATF-4 | NM_009716.3 | TGGGTTCTCCAGCGACAAGGC | GCATCCTCCTTGCCGGTGTCT |
| BMP1 | NM_001360021.1 | CCATATCCAGTCTCCCAATTACC | GTGACGCTCAATCTCAAAGG |
| BMP2 | NM_007553.3 | CCAAGACACAGTTCCCTACAG | ACCATGGTCGACCTTTAGGAG |
| BMP4 | NM_001316360.1 | CATCACGAAGAACATCTGGAG | CATCACGAAGAACATCTGGAG |
| BMP8b | NM_007559.5 | ACTTTGACCTAACCCAGATCCC | TCTATGCTGTGCCCATCCTC |
| GDF6 | NM_013526.1 | AAAGAAGGGAAGATGCAGCG | AGAGATCGTCCAGTCCTCTG |
| FGFr1 | NM_010206.3 | GAGCATCAACCACACCTACC | TATTAACTCCAGCAGTCTTCAGG |
| FGF7 | NM_008008.4 | CACACCACTTCATGATGTCG | CCGTTTCGTCTTGAGTTGCT |
| FGF11 | NM_001362623.1 | CTCCTTCACCCACTTCAATCTG | ACTCCTTAAAGCGACACTCTG |
| MEGF9 | NM_172694.2 | GCACCATTGAATCTGGAGAG | CCTTCTAGGCATTTCTCGCA |
| VEGFA | NM_001025257.3 | ACGTCAGAGAGCAACATCACC | CTGTGCTGTAGGAAGCTCATCTC |
| VEGFB | NM_001185164.1 | GAATGCAGATCCTCATGATCCA | GAGTGGGATGGATGATGTCAG |
| CyclinA1 | NM_001305221.1 | TGAAGTAAGACACAGACCCA | TTCATATTTCGAAGCCAGGAG |
| CyclinA2 | NM_009828.3 | CCAGTGAACGTTAATGAAGTACC | TTCAAACTTCGAAGCTAGCAG |
| CyclinB1 | NM_172301.3 | AAGGTGCCTGTGTGTGAACC | GTCAGCCCCATCATCTGCG |
| CyclinB2 | NM_007630.2 | AGTTATTCCTAAAGCCAAGAGCC | GTTCTGAGGTTTCTTCGCCA |
| CyclinD1 | NM_007631.2 | CGTGGCCTCTAAGATGAAGGA | AGTTCCATTTGCAGCAGCTC |
| CDK2 | NM_016756.4 | CCTCATCAAGAGCTATCTGTTCC | CAGCCCAGAAGAATTTCAGGT |
| CDK4 | NM_009870.4 | GCAGTCTACATACGCAACAC | GTCAGCATTTCCAGTAGCAG |
